# The clinical presentations of ectopic biliary drainage into duodenal bulbus and stomach with a thorough review of the current literature

**DOI:** 10.1186/1471-230X-10-2

**Published:** 2010-01-12

**Authors:** Ulku Saritas, Altug Senol, Yucel Ustundag

**Affiliations:** 1 Department of Internal Medicine, Süleyman Demirel University School of Medicine, Isparta, 32040 Turkey; 2 Department of Internal Medicine, Karaelmas University, Medical School, Zonguldak, 67100, Turkey

## Abstract

**Background:**

Ectopic biliary drainage is a rare congenital anomaly on which we have scarce data in the current literature.

**Methods:**

The data were collected from the records of 400 endoscopic retrograde cholangio-pancreatography (ERCP). In this report, we present 10 cases (male/female: 9/1, mean age 54 years, range 38-74) with ectopic biliary openings into the duodenum and/or stomach diagnosed by endoscopic retrograde cholangio-pancreatography (ERCP).

**Results:**

In our series, the frequency of ectopic biliary drainage is 2% (10 out of 400 ERCPs). Recurrent attacks of cholangitis and complicated ulcer formation in the distal stomach and bulbar duodenum were the most common signs in the present series. The sites of ectopic biliary drainage were the stomach in 1 case, the duodenum bulbus in 7 cases and the postbulbar duodenum in 2 cases. Bulbar ulcer, deformed pylorus and bulbus were present in 7 cases, apical bulbar stricture in 2, gastric ulcer in 1, pyloroplasty and/or gastroenterostomy in 3 cases. One case had had previous bleeding episode. Some of them had undergone previous surgeries for gall-stone disease (cholecystectomy in 5 cases, bile duct operation in 3 cases) and ulcer complications (pyloroplasty/gastroenterostomy in 3 cases). ERCP revealed dilatation of the biliary tree and hook shaped distal choledochus in all cases, choledocholithiasis in 7 and Mirizzi syndrome in 1. Endoscopic balloon dilatations for gastric outlet obstruction, extraction of bile stones after balloon dilating the ectopic site, surgery for difficult cases with large bile duct stones or with gastric outlet obstruction were preferred methods in this series of patients.

**Conclusion:**

With this report, we have to remind that ectopic biliary drainage must be considered in the differential diagnosis when the clinician faces cases with gastric outlet obstruction due to peptic ulcer formation accompanied by cholangitis/cholestasis.

## Background

Though it is not rare to see patients with congenital anomalies of the gall bladder and the biliary tree, ectopic opening of bile ducts to the third and fourth parts of the duodenum, the pyloric channel, the bulbus and the stomach is very much unusual [1-5]. The cases with this anomaly face recurrent attacks of cholangitis due to increased rate of choledocholithiasis and gastric outlet obstruction due to duodenal ulcer formation induced by duodenogastric reflux of bile constituents [6-8]. In this report, we planned to share our experience on a series of patients with ectopic biliary drainage with regard to clinical presentations of these cases. We also provided clues about a fast evaluation, endoscopic retrograde cholangiopancreatography (ERCP) findings and contribution of other investigative methods to help achieve a correct diagnosis in the setting of ectopic drainage anomaly of the biliary tree.

## Method

Between years 2004-2008, 400 ERCP procedures undertaken by the same endoscopist were retrospectively evaluated focusing on cases with ectopic biliary drainage. This study was approved by the local ethics committee of Suleyman Demirel University and written informed consent was taken from each patient with this anomaly for the publication of data and figures. The past medical history about previous cholangitis attacks, hepatobiliary surgical history, physical findings, complaints on admission to hospital, blood counts, liver transaminases, bilirubin levels and cholestatic enzymes, sonographic and tomographic findings were reported. The diagnosis of cholangitis was made if there were elevated cholestatic liver enzymes, leukocytosis and fever. When the papilla of Vater could not be localized in the usual anteromedial aspect of duodenum at the second part, we withdrew the duodenoscope to find the exact site of ectopic orifice, trying to concentrate on the appearance of the major papilla. Failing that, a forward-viewing gastroscope was inserted. When a gastric outlet obstruction was seen, an 18 mm dilatation balloon was used to dilate the stricture, and cannulation was employed after finding the biliary and/or pancreatic orifice. When the two orifices were noted, we cannulized each of them and made a contrast examination. The criteria for a diagnosis of ectopic biliary drainage or inclusion criteria into this study were defined as follows: 1-Absence of papilla at the usual second and third duodenal segments, 2-Contrast injection could not reveal the biliary tree, 3-Absence of another orifice despite the presence of choledochoduodenostomy. Exclusion criteria were noted as 1-the existence of another biliary drainage orifice and the papilla at normal localization through which biliary and pancreatic channels were demonstrated on contrast studies (cases with choledochoduodenal fistula). 2- When we could not find another ectopic orifice in cases with a choledochoduodenostomy history.

We did not perform sphincterotomy in cases with choledocholithiasis accompanied by ectopic biliary drainage merely due to the high risk of perforation. Rather we chose dilatation with pyloric balloons in cases with small stones in the bile duct. If we detected big stones in the choledochus and the patient had gastric outlet obstruction, then we referred such patients to surgery.

## Results

### Demographic and clinical data

Ten patients (1 female 9 male, mean age 54, age range 38-74 years) with signs of cholangitis and/or extrahepatic cholestasis were diagnosed as having ectopic biliary drainage anomaly on ERCP procedure. All the patients, except one, were symptomatic. Seven cases had abdominal pain, 7 had jaundice and 4 had fever. The only asymptomatic case had cholangitis attacks a few times in the last 1-2 years and he was referred to us for investigation of the cause of these attacks.

The case with ectopic bilio-pancreatic drainage into the antrum had gastric ulcer which bled 2 weeks before his admission. At that time, he had been administered 7 units of blood transfusion. Five patients had undergone cholecystectomy previously. One case had been treated with choledocho-duodenostomy due to recurrent cholangitis attacks. One patient had developed biliary fistula to the abdominal space and had to be treated with several tube drainages for some period of time. Another patient had undergone an unknown type of operation involving biliary tree approximately 4 years after his cholecystectomy operation. All these patients, except one, had elevated cholestatic enzymes and direct hyperbilirubinemia. Five cases had leukocytosis. Rest of the clinical data of all the patients is summarized in Table 1.

**Table 1:** The demographic, clinical data and endoscopic findings about our cases are presented.

Patient no	Age	Complaints	Duration of symptoms	CS	Bile duct operation	Gall stone	Bile duct stone	Ectopic biliary drainage to	Duodenal ulcer/Gastric ulcer	Apical stricture	Gastric operation	Endoscopic treatments (EST, EBD)	Stent	Stone extraction	Surgery
**1**	75	Jaundice	1.5 months	Yes	No	Yes	No, but Mirizzi	Post-bulbar	No/No	No	No	EST	Yes	No	CS, HJ
**2**	54	Jaundice	5 months	No	Yes	Operated	Yes	Bulbus	Yes/No	Yes	Yes, PP	No	No	No	Exitus
**3**	45	Cholangitis	9 years	No	No	Yes	No	Bulbus	Yes/No	No	No	No	No	No	No
**4**	58	Malaise	10 years	Yes	Yes	Operated	No	Post-bulbar	Yes/No	No	No	No	No	No	No
**5**	74	Abdominal pain	2 months	Non	No	Yes	Yes	Stomach	No/No	No	No	EBD	No	Yes	CS, HJ
**6**	49	Abdominal pain, vomiting, fever	4 years	Yes	Yes	Operated	Yes	Bulbus	Yes/No	No	Yes, GJ	EBD	No	Yes	No
**7**	38	Abdominal pain	2 months	Yes	No	Operated	Yes	Bulbus	Yes/No	No	No	EBD	Yes	Yes	No
**8**	53	Cholangitis	7 months	Yes	No	Operated	No	Bulbus	No/No	No	No	No	No	No	No
**9**	49	Fever	5 months	No	No	Yes	Yes	Bulbus	Yes/No	No	Yes, GJ	EBD	No	Yes	No
**10**	45	Jaundice	1.5 months	No	No	Yes	Yes	Bulbus	Yes/No	Yes	No	EBD	Yes	Yes	CS, HJ

PP: Pyloroplasty, GJ: Gastrojejunostomy CS: Cholecystectomy HJ: Hepaticojejunostomy EST: Endoscopic sphincteroplasty EBD: Endoscopic biliary dilatation

### Endoscopic findings

**Figure 1:**
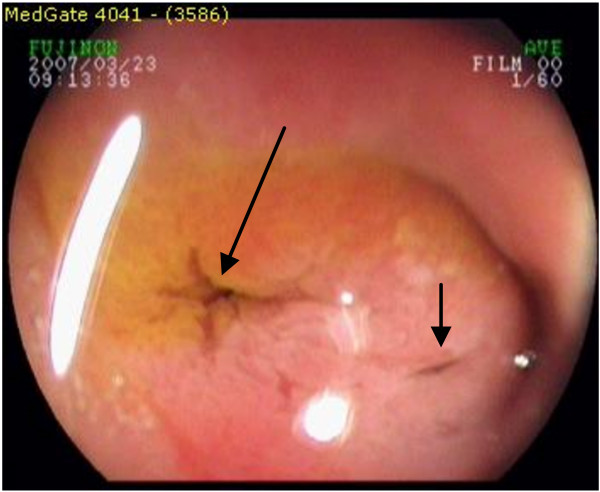
**A case with ectopic bilio-pancreatic drainage into the stomach**. Biliary orifice is indicated by taller arrow. The pancreatic duct orifice is shown by smaller arrow. It is clear to see small amount of bile staining the biliary orifice.

**Figure 2:**
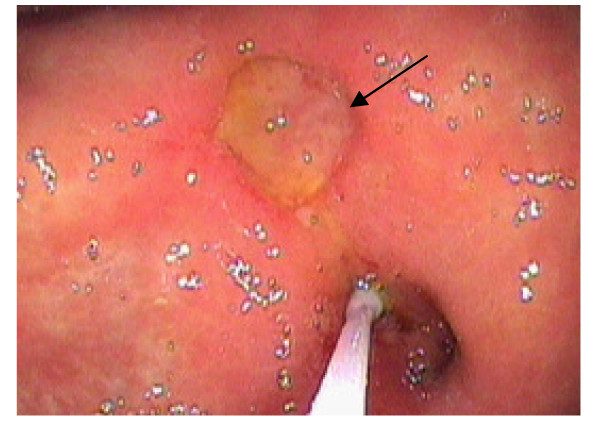
**Associated gastric abnormalities**. Large antral ulcer is indicated by an arrow. We also see the ERCP catheter placed in the bile duct through the orifice in the stomach.

Four patients had 2 different orifices in bulbus, whereas four had one orifice. Four cases had drainages to the anterior bulbar wall, three patients had opening to the apical part. Two patients had papilla vateri located at the posterior part of postbulbar duodenum. In a patient with biliopancreatic drainage into prepyloric antrum, there were 2 orifices in different sizes and a 1 cm ulcer with a fibrotic appearance (Figure 1 and 2). Endoscopic findings are clearly demonstrated in Table 1.

### ERCP findings

**Figure 3:**
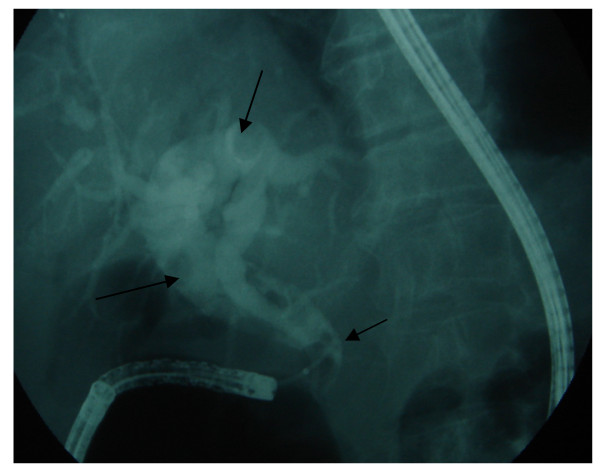
**ERCP appearance**. Dilated biliary tree and choledocholithiasis on cholangiography (long arrows). Distal end of choledochus is seen as hook shaped structure (short arrow).

**Figure 4:**
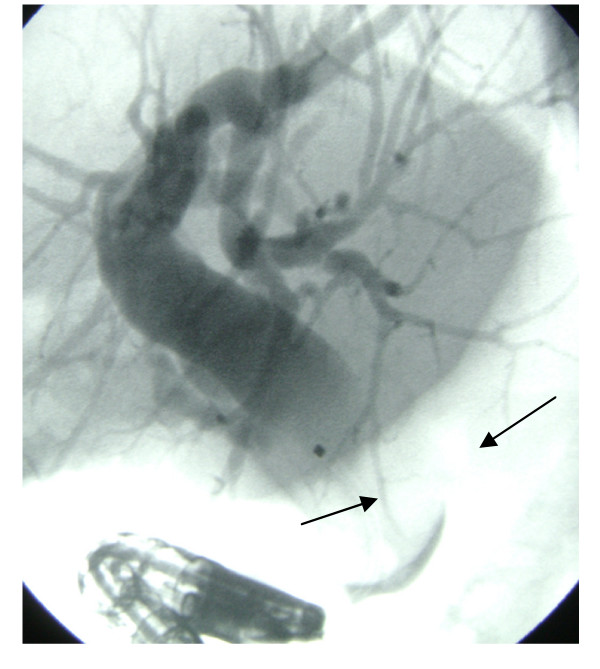
**ERCP appearance**. Dilated choledochus and unclaved bile duct stone (nearly 2.5 cm in size) at distal end (arrows).

The choledochus and intra-hepatic biliary tree were found to be dilated in all patients with ectopic drainage anomaly and distal end of choledochus emptied into the duodenum with a hook shape configuration. Five patients had 2 different ectopic orifices of biliary and pancreatic ducts (one case had both channels' openings into the antrum) through which both channels were visualized by contrast examination. Only the bile ducts were identified on contrast examinations in 5 cases there being no absolute reason to demonstrate the pancreatic channel. Seven cases had bile duct stones and 2 of them had stones bigger than 2 cm. These big stones had been enclaved on the hook shaped distal end of choledochus (Figure 3, 4). Three patients did not have bile duct stones. One of them had undergone several biliary surgical procedures due to recurrent cholangitis attacks since 1987. In other centers, ERCP had been tried several times in this patient and the endoscopists could not show papilla and failed at each trial. This patient had ectopic papilla and choledochoduodenostomy opening at bulbus. At the junction of choledochus and cystic channel, the pressure due to stone impacted in Hartman pouch led to type IV Mirizzi syndrome.

### Other imaging methods

Abdominal ultrasonography performed in each patient showed dilated biliary tree in all of them. Besides, five had choledocholithiasis, 4 had hydropic gall bladder containing small stones on ultrasonography. One patient also had air in intrahepatic bile ducts. Another case had signs of obstructive jaundice and his past medical history was positive for peptic ulcer disease. Abdominal ultrasonography showed dilated stomach fully filled with large amount of fluid. The choledochus was 2.5 cm in diameter and also contained approximately a 2.5 cm-sized bile stone. An ectopic biliary opening and gastric outlet obstruction were presumed to be part of differential diagnosis in this case and were later confirmed by upper gastrointestinal endoscopy.

**Figure 5:**
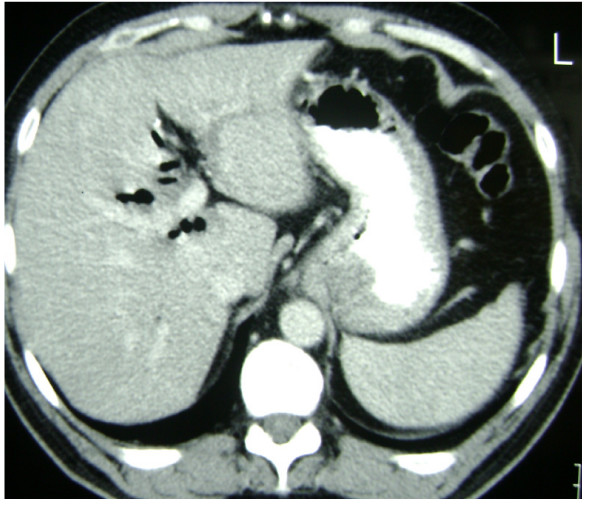
**CT appearance**. Pneumobilia in CT.

**Figure 6:**
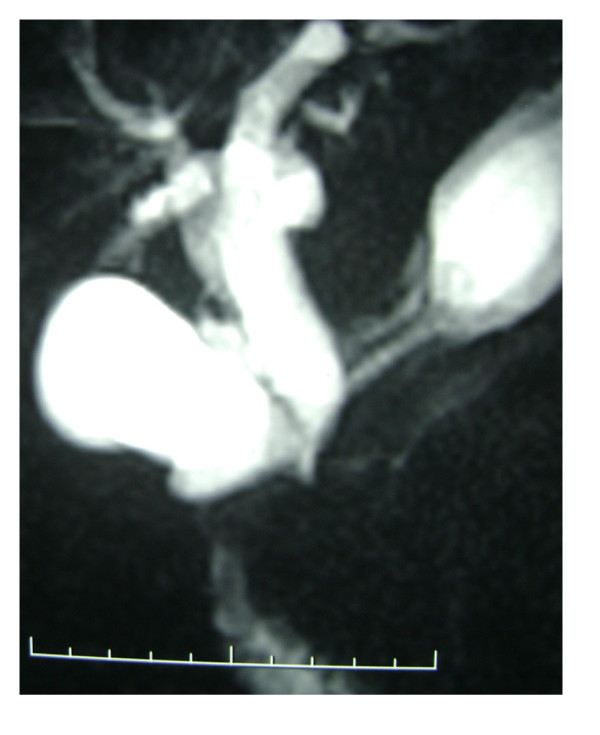
**MRCP appearance**. An MRCP examination reveals dilated whole biliary tree and hook shaped distal choledochus. MRCP did not delineate bile duct stones.

On abdominal CT examination, five patients had dilated choledochus, 3 had stones in the common bile duct and one had air in the biliary tree (Figure 5). On MRCP, one patient had small stones in the common bile duct and the other had dilated biliary tree which terminated into the duodenum in a hook-like appearance (Figure 6).

### Treatment

**Figure 7:**
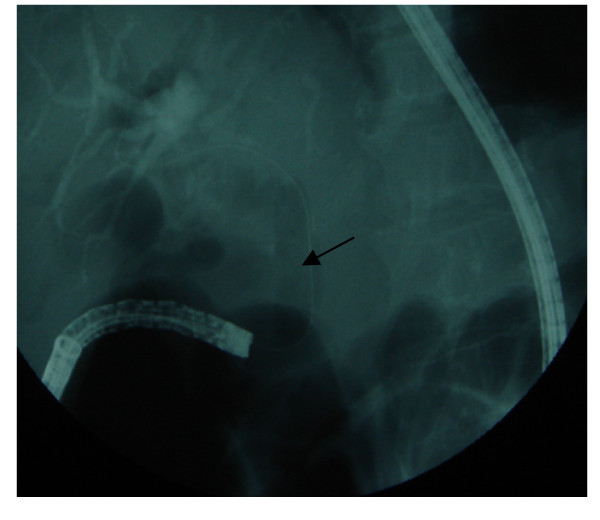
**Therapeutic endoscopy. **Dilatation of biliary orifice with pyloric dilatation balloon (arrow) in a case with ectopic drainage of biliary tree into the stomach and choledochus.

**Figure 8:**
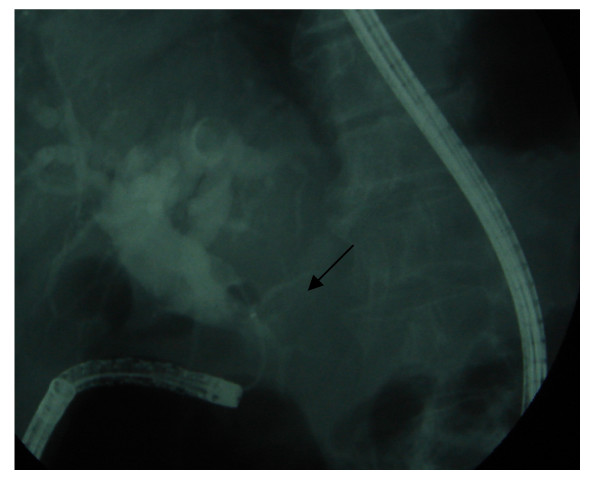
**Therapeutic ERCP**. Stone extraction after biliary balloon dilatation (arrow indicate stone extractor balloon)

All patients received supportive medical treatment modalities. The signs of cholangitis regressed only after medical treatment in 2 cases. Stone extraction from choledochus was performed in 3 cases after dilating the biliary orifice with pyloric and/or biliary balloon dilators (Figure 7 and 8). We inserted a plastic stent into the biliary tree due to inability to remove a large common bile duct stone in one patient. Later on, this patient underwent surgical therapy. Another patient with ectopic opening into the stomach underwent cholecystectomy and choledocho-duodenostomy due to cholecystitis and recurrent choledochal stone disease, 6 months after endoscopic clearance of biliary stones. We proposed surgical therapies for the rest of cases (3 patients) due to unsuccessful attempts for stone clearance on ERCP. One of them died due to cardiac arrhythmia just before the operation (11%). The other 2 cases underwent surgical therapy including pyloroplasty, cholecystectomy and Roux-en-Y gastroduodenostomy. A case with Mirizzi syndrome and ectopic biliary drainage to postbulbar duodenum was treated with plastic stent insertion up to liver hilum. Afterwards, surgery was performed in this case. In brief, 3 patients were treated by surgery and 3 by endoscopic methods, 2 by both methods, and 2 only by medical agents. Unfortunately, we do not have a long term follow up data for our cases.

## Discussion

At times, the papilla of Vater can terminate at aberrant sites, including the stomach, pyloric canal, duodenal bulb, and the third or fourth portion of the duodenum. This is named as ectopic biliary drainage and it is a rather rare condition. Although, we do not know the exact frequency of ectopic biliary drainage, the frequency of the ectopic biliary drainage was found to be 2% in our series. In 2001, Bernard P et al. reported only 23 cases with this condition by then [9]. In the literature, there were some other small case series and when we analyzed all these cases, we noticed that they were mostly patients with double choledochus [9-12]. Lee and et al from Korea indicated 18 cases with this anomaly out of 16541 ERCP records [8]. The largest case series in the literature was published by Disibeyaz and et al from Turkey reported 53 patients (0.43%) with ectopic biliary drainage out of 12158 ERCP records [13]. In these big series of patients with this anomaly, there was no record of any case with ectopic drainage into the stomach. Lindler and et al. reviewed their 1000 intraoperative cholangiograms and reported that ectopic biliary drainage distal to the second part of the duodenum was present in 13.1% of the cases [14]. However, we believe that it is insufficient to evaluate only cholangiograms and, diagnostic potential of endoscopy visualizing the exact site or origin of biliary drainage into the duodenum or stomach seems to be superior to operative cholangiograms.

### Etiology and classification

Though we do not know its etiology, it must be due to an abnormality during the embryonic developmental period. For up to 8 weeks of gestation, the extra-hepatic biliary tree develops through lengthening of the caudal part of the hepatic diverticulum. The hepatic duct (ductus hepaticus) develops from the cranial part (pars hepatica) of the hepatic diverticulum. The distal portions of the right and left hepatic ducts develop from the extrahepatic ducts and are clearly defined tubular structures by 12 weeks of gestation. The proximal portions of the main hilar ducts derive from the first intrahepatic ductal plates. In the first weeks of embryogenesis, if subdivision occurs very early, leaving the pars hepatica above the zone of growth that separates stomach from duodenum, then the pars hepatica will develop into a duct emptying into the region of the pylorus [7]. Very rarely, two choledochus with 2 different opening orifices into the duodenum can be seen [15]. However mostly, the choledochus which would open into the duodenum at its usual position becomes rudimentary and the other one with a fully developed channel drain into an ectopic opening in the stomach or duodenum.

Kanematsu et al. proposed the classification of the congenital anomalies associated with ectopic drainage of the biliary tract in the following 4 types [11]:

**Type I: **There is one septated draining duct, which enters the stomach.

**Type II: **Division of the common bile duct in 2 separate junctions, one of which enters the normal position of the papilla of Vater, whereas the second one enters the stomach.

**Type III: **Presence of 2 independent draining ducts, one of which enters the normal position of the papilla of Vater, whereas the second one enters the stomach.

**Type IV: **Presence of 2 independent draining ducts, one of which enters the normal position of the papilla of Vater, whereas the second one enters the stomach and share one or more intercommunications.

The most common location of the ectopic biliary drainage into the stomach is the corpus of the stomach, followed by the antrum and cardia. One of our cases had ectopic biliary and pancreatic channel drainage into gastric antrum. Ectopic biliary drainage into the third and fourth part of the duodenum was not present in our series. These conditions have been reported to associate with pancreatic divisum and long common channel anomaly [16,17]. There has been no ulcer formation in ectopic biliary drainage anomaly in the distal duodenum. In our case with postbulbar ectopic drainage condition, he had no complaints of ulcer disease. This may be due to the resistance of postbulbar duodenal mucosa to bile and duodenal peristaltism helps this by decreasing bile contact with duodenal mucosa.

### Demographics and clinics

In the literature, more than 80% of the cases with ectopic biliary drainage into the bulbar duodenum were reported to be the male gender. In our series, 90% of the patients had male gender. This might bring to mind the etiologic relation with Y chromosome-induced embryonic abnormality. However, this condition can also be seen in female patients. Proximal ectopic biliary drainage seemed to be common in middle-aged patients and distal ectopic opening was commonly encountered in pediatric population. Our patients were over 50 years of age when they were diagnosed with ectopic biliary drainage. These cases became symptomatic at the 4th or 5th decades of life with signs and symptoms of cholangitis and upper gastrointestinal bleeding. Due to absence of a sphincteric structure, chronic regurgitation of intestinal contents into the biliary tree leads to recurrent cholangitis attacks, biliary stone formation and distal biliary stricture [6-8]. Bile reflux into the duodenal bulbus and the stomach causes ulcer formation and its associated complications [6-8]. In the literature, 2 cases with ectopic biliary drainage were diagnosed with gastric cancer and precancerous changes around the ectopic biliary opening led to theories indicating possible relationship with gastric carcinogenesis. In summary, 80% of the patients had ulcer and ulcer-related complications.

Except for one case who developed cholangitis one week before his admission to our clinic and treated with medical methods, all had complaints due to biliary disease. The main indication for ERCP procedures undertaken in all cases was the presence of biliary symptoms and laboratory abnormalities. In our series, there was no case with incidental diagnosis of ectopic biliary drainage.

### Diagnosis

The upper endoscopy and ERCP provide us with necessary information about this condition with detection of ectopic orifice and cholangiographic appearance. When the papilla vateri is not located at its original place in the second part of duodenum and moreover if there is associated ulcer formation, we have to think of ectopic biliary drainage anomaly. It is difficult to conduct a duodenoscopic examination in most of the patients with ectopic biliary drainage abnormality due to ulcer-related pyloric and duodenal deformity. In such cases, forward viewing gastroscopes can reveal the bulbus easily. Ectopic orifice, usually stained with bile, is a slit-like opening sized 2-3 mm. If there is no bile on it, an aspiration maneuver can reveal bile drainage. There is no classic papillary appearance in these cases. However, Kubota et al. indicated papillary-like protuberance in the duodenum bulbus in 2 cases [18]. On ERCP examination, distal choledochus has a hook-like appearance and is larger and shorter than normal choledochus. In some cases, there may be a short stricture at distal choledochus. Mostly these cases had dilated left intrahepatic biliary tree and occasionally, there are bile duct stones.

Less invasive or even non-invasive methods can be used for the diagnosis of this condition. Absence of the sphincteric muscle leads to pneumobilia in these cases. Pneumobilia can also be seen in the presence of choledochoduodenal fistula and choledochoduodenostomy. Thus, it is not specific for cases with ectopic biliary drainage problem. Some authors also propose that spontaneous passage of barium into the biliary tree support the diagnosis of ectopic biliary drainage problem. However, a similar finding can also be seen in cases with choledochoduodenostomy.

In the literature, we could not find any data about additional contribution of abdominal ultrasonography to the diagnosis of ectopic biliary drainage. An abdominal ultrasound examination showed dilated stomach in one of our cases admitted for obstructive jaundice. In this case, choledochus was 2.5 cm in maximal diameter and contained similarly large-size stones within its lumen. Though the choledochus was dilated but not tortuosed in appearance, we thought it to be shorter than its normal size. Gastric dilatation was attributed to gastric outlet obstruction due to bulbar ulcer. Thus, abdominal ultrasonography helped us to make an unequivocal diagnosis of ectopic biliary drainage. In the literature, there are case reports favoring the utility of endoscopic ultrasound examination and MR cholangio-pancreatography in the diagnosis of ectopic biliary drainage problem [10,19-21]. In a case series of 8 patients with ectopic biliary drainage anomaly, computerized tomography could not reveal any specific finding [21].

### Treatment

**Figure 9:**
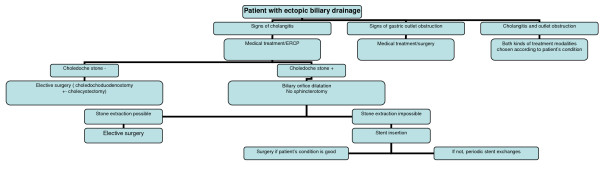
**A therapeutic flow-chart**. An algorithm of practical approach to cases with ectopic biliary drainage is provided.

In routine clinical practice, the treatment of ectopic biliary drainage is towards biliary symptomatology and associated peptic ulcer complications [8,13]. Gastric outlet obstruction can be handled with endoscopic balloon dilatations and/or surgical bypass methods. If there is ongoing cholangitis, physicians should treat cholangitis and also take some precautions to protect the patient against recurrent cholangitis attacks. The first-line treatment modality should be endoscopy in these patients as surgical treatments carry appreciable risks for them due to different anatomical composition. In patients with ectopic drainage problem, the entry site for choledochus to duodenum is a slit-like opening without a sphincteric structure and there is no intramural part of choledochus in these cases. Thus, sphincterotomy carries an appreciable difficulty and risk of retroduodenal perforation in such cases. Most authors advise extraction of bile stones after balloon dilating of ectopic opening site. If the case is elderly and stones cannot be extracted, then plastic biliary stents can be introduced after biliary drainage. Moreover, difficult stones in cases with gastric outlet obstruction can be managed best with surgery. It is generally agreed that these patients should undergo frequent endoscopic controls for early recognition and treatment of the above-mentioned complications. Some surgeons propose the resection of the common bile duct combined with hepaticojejunostomy to ameliorate the possible danger for the development of gastric adenocarcinoma. Other conservative approaches include the ligation of the ectopic draining duct (in cases of double opening) or the reinsertion of the ectopic bile duct in the duodenum [10,11,18]. However, informing the surgeon of the current anatomic problem in these cases would be of crucial importance to lower the risks in surgery. Based on our experience in our cases, we suggested a therapeutic flow-chart of ectopic biliary drainage (Figure 9).

## Conclusion

In conclusion, ectopic biliary drainage should be taken into consideration in cases having gastric outlet obstruction due to peptic ulcer formation accompanied by cholangitis/cholestasis. This anomaly should be known by endoscopists who participate in ERCP procedures. It should be kept in mind that when there is no papilla in its normal anatomic site, ectopic biliary drainage can be an alternative diagnosis and bile stained slit-like biliary orifice should be investigated in the first, third or fourth parts of the duodenum. This knowledge is especially important for surgeons who will take care of these patients for operations of the gastric and biliary system. Thus, serious biliary injuries can be prevented by sharing this information with surgeons.

## Competing interests

The authors declare that they have no competing interests.

## Authors' contributions

US carried out ERCP procedures and has made substantial contributions to conception and design, analysis and interpretation of ERCP data; she was also involved in drafting the manuscript critically for important intellectual content. She had given final approval of the version to be published. YU provided great amount of contribution and effort to analysis and interpretation of ERCP data. He participated actively in drafting the manuscript and revising it critically for important intellectual content. He had given final approval of the version to be published. AS provided important contributions to design the paper, and acquisition and analysis of data. He participated in drafting the manuscript. He had given final approval of the version to be published.

## Pre-publication history

The pre-publication history for this paper can be accessed here:

http://www.biomedcentral.com/1471-230X/10/2/prepub
